# HSD11B1 is upregulated synergistically by IFNγ and TNFα and mediates TSG-6 expression in human UC-MSCs

**DOI:** 10.1038/s41420-020-0262-7

**Published:** 2020-04-20

**Authors:** Peiqing Huang, Yinghong Li, Chenchang Xu, Gerry Melino, Changshun Shao, Yufang Shi

**Affiliations:** 1 State Key Laboratory of Radiation Medicine and Protection, Institute for Translational Medicine, Key Laboratory of Stem Cells and Medical Biomaterials of Jiangsu Province, Medical College of Soochow University, Suzhou, China; 2 Department of Experimental Medicine, TOR, University of Rome Tor Vergata, Rome, Italy; 3 Medical Research Council (MRC) Toxicology Unit, University of Cambridge, Cambridge, UK; 4 The First Affiliated Hospital of Soochow University, Suzhou, China; 5 Key Laboratory of Tissue Microenvironment and Tumor, Shanghai Institutes for Biological Sciences, Chinese Academy of Sciences, Shanghai, China

**Keywords:** Mesenchymal stem cells, Cytokines

## Abstract

Inflammatory factors such as IFNγ and TNFα could endow mesenchymal stem cells (MSCs) a potent immunomodulatory property, a process called licensing, but the mechanisms are not fully understood. We here found that glucocorticoid-activating enzyme 11β-hydroxysteroid dehydrogenase type 1 (HSD11B1), which converts inactive cortisone to the active cortisol and thereby regulates tissue glucocorticoid (GC) levels, was greatly upregulated by IFNγ and TNFα in human umbilical cord-derived MSCs (UC-MSCs) in a synergistic manner. While IFNγ alone was not able to induce HSD11B1, it could increase the activity of NF-kB and thus augment the upregulation of HSD11B1 by TNFα. Interestingly, the upregulation of HSD11B1 by IFNγ and TNFα also required glucocorticoid receptor. Furthermore, HSD11B1 was shown to be required for the expression of TNF-stimulated gene 6 (TSG-6), an important anti-inflammatory effector molecule of MSCs. Therefore, the inflammatory factors IFNγ and TNFα can promote GC metabolism and thereby drive the expression of anti-inflammatory factor TSG-6 in human UC-MSCs, forming a potential negative feedback loop. These findings help to understand the relationship between inflammation and GC metabolism.

## Introduction

HSD11B1 (11β-hydroxysteroid dehydrogenase type 1), a luminally oriented enzyme of the endoplasmic reticulum membrane^1^, is wildly expressed in the body. It functions to convert inactive cortisone to the active cortisol^2^. Because endogenous glucocorticoid (GC) is indispensable for maintaining homeostasis, HSD11B1 is also expected to play a critical role in the modulation of metabolism and inflammatory response. Indeed, some chronic inflammatory conditions have been found to be associated with increased HSD11B1 expression, such as atherosclerosis^3^, inflammatory bowel disease, and colitis^4,5^. These observations are in line with many reports of induction of HSD11B1 expression by proinflammatory cytokines in various cell types^6–8^. In general, the regulation of HSD11B1 expression by inflammatory factors appears to be cell type-specifics. For instance, TNFα induce HSD11B1 expression in human fibroblasts, but not in human primary hepatocytes^9,10^. Therefore, the expression and function of HSD11B1 remain to be further elucidated.

Various studies have demonstrated the exciting therapeutic effect of MSCs in multiple sclerosis^11^, GvHD^12^, fibrosis^13^, systemic lupus erythematosus (SLE)^14^, acute lung injury^15^, inflammatory bowel diseases^16^. And there are many ongoing Phase I–III clinical trials of MSCs in treating inflammatory disease^17^. However, it should be noted that MSCs are plastic in their immunomodulation^18^. The immunosuppressive function of MSCs could be reversed by GC when the two combined to aggravate inflammation in mice^19^, which reflects the complex interaction between GC and MSCs.

Published investigations have shown that the therapeutic effect of MSCs is mainly a result of immunomodulation and that this function is licensed by inflammation^12^. A cocktail of proinflammatory cytokines, IFNγ in combination with any of three other cytokines, TNFα, IL-1α, or IL-1β, was shown to have the capability to induce the immunosuppressive function of MSCs^12^. The licensed MSCs were found to secrete several factors and metabolites that mediate the anti-inflammatory effect of MSCs.

TSG-6, a hyaluronan-binding protein, is one of the factors secreted by human MSCs. It can attenuate inflammation and enhance tissue repair in mouse models of acute lung injury^15^, peritonitis^20^, myocardial infarction^21^, corneal injury^22^, and arthritis^23^. However, despite the well-recognized role of TSG-6 in immunomodulation, the regulation of TSG-6 expression in human MSCs is not very clear. We previously reported that while TNFα could induce TSG-6 expression in human MSCs, the induction could be substantially enhanced by another cytokine IFNγ, via kynurenic acid, a metabolite of indoleamine 2,3-dioxygenase (IDO)^24^.

In the present study, we found that HSD11B1 was synergistically upregulated by IFNγ and TNFα in human umbilical cord-derived MSCs (UC-MSCs). In this process, IFNγ was found to promote the phosphorylation of p65, a NF-κB subunit, and thus augment the HSD11B1 expression induced by TNFα. To our surprise, glucocorticoid receptor (GR) was activated by IFNγ and TNFα and mediated the upregulation of HSD11B1 by IFNγ and TNFα. We also identified HSD11B1 to be an essential molecule for the induction of anti-inflammatory TSG-6 by IFNγ and TNFα. Our study reveals a novel link between inflammatory factors, HSD11B1 and TSG-6 in human UC-MSCs. This finding increases our understanding of the regulation and function of HSD11B1 in inflammation, and may contribute to optimization of MSCs-based clinical treatments for inflammatory conditions.

## Results

### HSD11B1 was synergistically upregulated by IFNγ and TNFα in human UC-MSCs

**Fig. 1:**
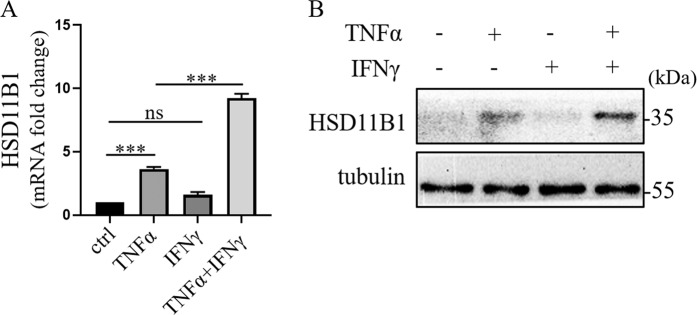
The expression of HSD11B1 was significantly increased in IFNγ and TNFα licensed human UC-MSCs. **a** Real-time PCR was employed to measure HSD11B1 in human UC-MSCs after TNFα and IFNγ stimulation. The histograms represent the mean values ± SEM from three independent experiments (*n* = 9). Asterisks indicate highly significant (***p < 0.001) differences (Student’ *t* test). Data are presented as fold change relative to ctrl human UC-MSCs. **b** Western blotting was performed to detect HSD11B1 in human UC-MSCs after TNFα and IFNγ stimulation.

Previous reports have shown that the immunosuppressive function of MSCs is induced by proinflammatory cytokines, especially the combination of IFNγ and TNFα. However, most of the previously identified anti-inflammatory functions of MSCs are based on the secretory effector molecules, such as TSG-6 and IGF-2^11^. Because GC is critically involved in the regulation of inflammation, we set to explore the roles of GC metabolism in MSCs. Several reports showed that HSD11B1, the main enzyme that metabolizes GC, was upregulated in human fibroblasts, chondrocytes, and adipocytes by TNFα^25^. We therefore first examined the direct effect of IFNγ and TNFα stimulation on HSD11B1 expression in human UC-MSCs. We added recombinant human TNFα and IFNγ each alone or in combination to the culture of human UC-MSCs. TNFα induced the expression of HSD11B1 (Fig. 1a, b), which is consistent with other studies. Surprisingly, while IFNγ alone did not induce HSD11B1, IFNγ, and TNFα in combination substantially increased the expression of HSD11B1. These results showed that IFNγ and TNFα could synergistically upregulate the expression of HSD11B1 in human UC-MSCs.

### NF-κB mediates the synergistic effects of IFNγ and TNFα on HSD11B1 expression

**Fig. 2:**
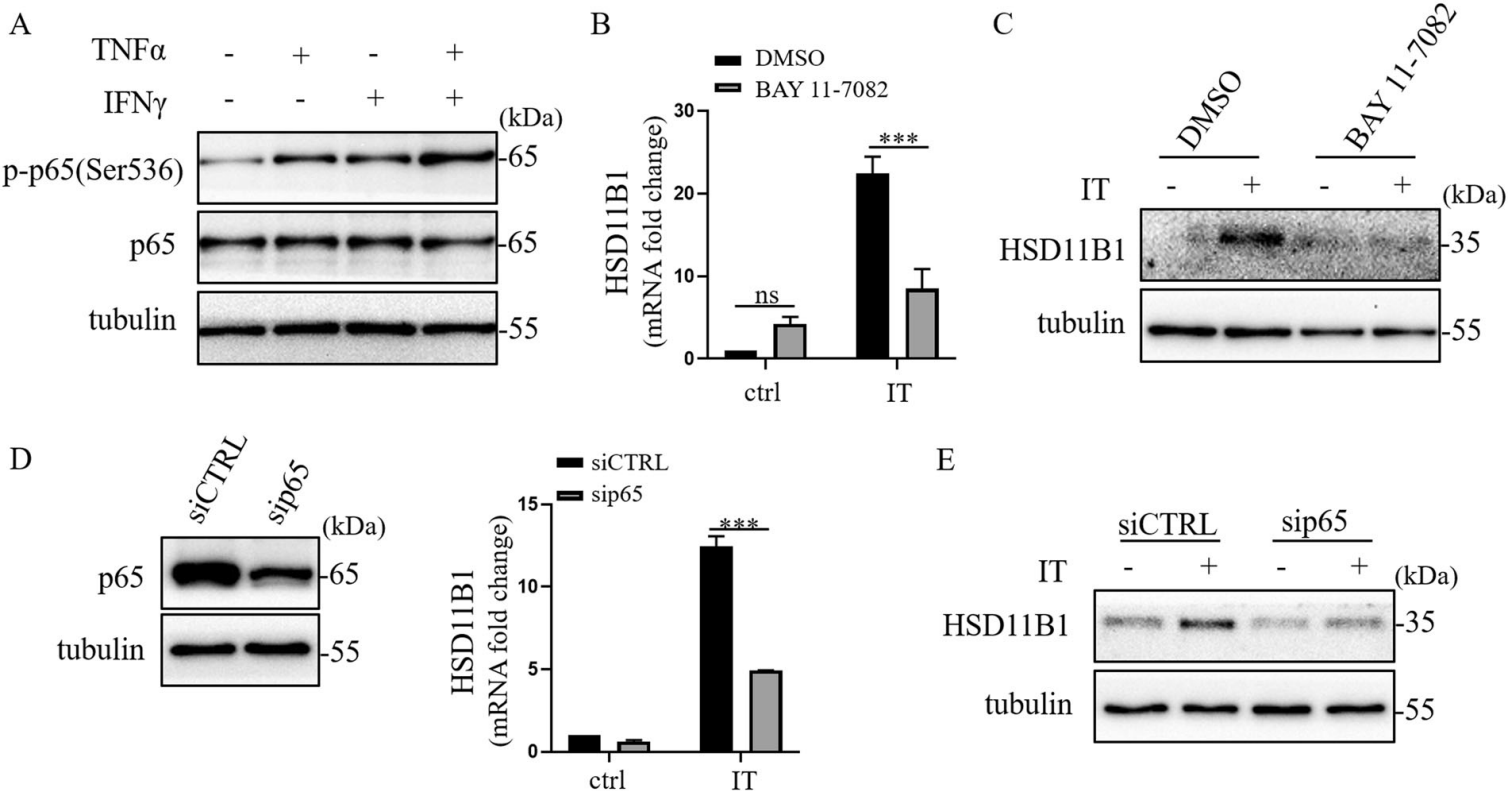
IFNγ and TNFα synergistically enhanced the expression of HSD11B1 in human UC-MSCs via NF-κB p65. **a** Western blotting was performed to detect p-p65 (Ser536) in human UC-MSCs after TNFα and IFNγ stimulation. **b** Real-time PCR was employed to measure HSD11B1 in human UC-MSCs when NF-κB was inhibited by BAY 11-7082 in IT (TNFα combined with IFNγ) stimulation. The histograms represent the mean values ± SEM from three independent experiments (*n* = 9). Asterisks indicate highly significant (***p < 0.001) differences (ANOVA). Data are presented as fold change relative to DMSO-ctrl human UC-MSCs. **c** Western blotting was performed to detect HSD11B1 in human UC-MSCs when NF-κB was inhibited by BAY 11-7082 in IT stimulation. **d** Knockdown efficiency of sip65 was examined by Western blotting. Real-time PCR was employed to measure HSD11B1 in p65-depleted human UC-MSCs after IT stimulation. The histograms represent the mean values ± SEM from three independent experiments (*n* = 9). Asterisks indicate highly significant (***p < 0.001) differences (ANOVA). Data are presented as fold change relative to siCTRL-ctrl human UC-MSCs. **e** Western blotting was performed to detect HSD11B1 in p65-depleted human UC-MSCs after IT stimulation.

Since TNFα mainly acts through NF-κB signaling in regulating downstream genes pathway, we determined the phosphorylation level of p65, a NF-κB subunit, in human UC-MSCs in the presence of TNFα and IFNγ each alone or in combination. We observed that either of the two cytokines could increase the level of p-p65, but the combination resulted in the highest p65 phosphorylation level (Fig. 2a).

To determine the role of p65 in HSD11B1 regulation, we examined the effect of BAY 11-7082, an NF-κB inhibitor, on HSD11B1 expression in IFNγ and TNFα -treated human UC-MSCs. The results showed that the upregulation of HSD11B1 by the cytokines was nearly abolished when NF-κB activity was inhibited (Fig. 2b, c). To further investigate this phenomenon, we knocked down p65 in human UC-MSCs by siRNA, and found that the expression of HSD11B1 was suppressed after p65 depletion (Fig. 2d, e). These results indicate that the upregulation of HSD11B1 by IFNγ and TNFα was achieved through NF-κB signaling.

### GR was required for the upregulation of HSD11B1 by IFNγ and TNFα

**Fig. 3:**
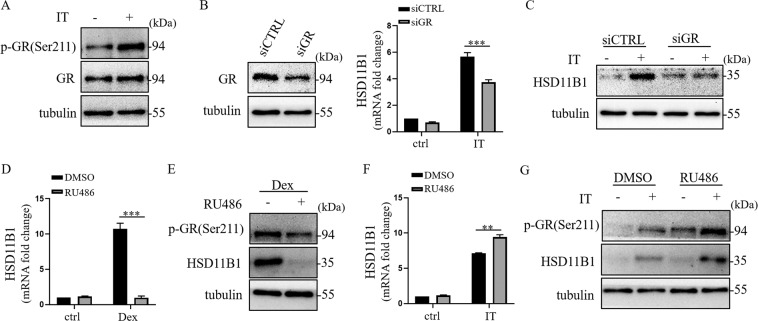
Glucocorticoid receptor (GR) was involved in IFNγ and TNFα-induced HSD11B1 expression. **a** Western blot analysis was performed to detect p-GR(Ser211) in human UC-MSCs under IT (TNFα combined with IFNγ) stimulation. **b** Knockdown efficiency of GR was examined by Western blotting. Real-time PCR was employed to measure HSD11B1 in GR-depleted human UC-MSCs after IT stimulation. The histograms represent the mean values ± SEM from three independent experiments (*n* = 9). Asterisks indicate highly significant (***p < 0.001) differences (ANOVA). Data are presented as fold change relative to siCTRL-ctrl human UC-MSCs. **c** HSD11B1 was detected in GR-depleted human UC-MSCs after IT stimulation by Western blotting. **d** Real-time PCR was performed to detect HSD11B1 in human UC-MSCs under stimulation of dexamethasone or RU486 alone or a combination of them. The histograms represent the mean values ± SEM from three independent experiments (*n* = 9). Asterisks indicate highly significant (***p < 0.001) differences (ANOVA). Data are presented as fold change relative to DMSO-ctrl human UC-MSCs. **e** Western blotting was employed to detect HSD11B1 and p-GR(Ser211) in human UC-MSCs under stimulation of dexamethasone or RU486 alone or a combination of them. **f** Real-time PCR was employed to measure HSD11B1 in human UC-MSCs under stimulation of RU486 alone or a combination of IT. The histograms represent the mean values ± SEM from three independent experiments (*n* = 9). Asterisks indicate highly significant (**p < 0.01) differences (ANOVA). Data are presented as fold change relative to DMSO-ctrl human UC-MSCs. **g** Western blotting was employed to detect HSD11B1 and p-GR(Ser211) in human UC-MSCs under stimulation of RU486 alone or a combination of IT.

It was well established that GCs can upregulate HSD11B1 expression in a variety of cells by activating GR. In addition, phosphorylation at the Ser211 site was reported to be a marker of GR activation^26^. To test whether GR is involved in the upregulation of HSD11B1 by the two cytokines, we examined the phosphorylation level of GR in human UC-MSCs exposed to IFNγ and TNFα stimulation. We found that the level of phosphorylated GR at Ser211 was significantly higher in the cytokines-treated cells than in control (Fig. 3a).

To further explore the role of GR in IFNγ and TNFα-induced HSD11B1 expression, we knocked down GR in human UC-MSCs by transfection with siRNA. Interestingly, after GR depletion, the upregulation of HSD11B1 by IFNγ and TNFα was weakened (Fig. 3b, c), suggesting that GR is involved in the regulation of HSD11B1 by IFNγ and TNFα.

We next treated human UC-MSCs with RU486, a GC competitive GR inhibitor. As expected, RU486 greatly inhibited the induction of HSD11B1 by dexamethasone (Dex) (Fig. 3d, e). However, to our surprise, RU486 was found to promote the expression of HSD11B1 induced by IFNγ and TNFα at both mRNA and protein levels (Fig. 3f, g). At the same time, level of p-GR(Ser211) increased. While RU486 inhibited the p-GR(Ser211) induced by Dex (Fig. 3e), it increased the level of p-GR(Ser211) induced by IFNγ and TNFα (Fig. 3g). These results indicated that while RU486 can competitively inhibit the effect of Dex, it does not impair the function of GR in promoting HSD11B1 expression in the context of IFNγ and TNFα-treated human UC-MSCs. This result suggest that GR probably played a structural role in IFNγ and TNFα-licensed human UC-MSCs. Future studies are required to determine whether GR functions to coordinate the binding of NF-kB and other transcription factors to HSD11B1 promoter.

### HSD11B1 is required for the induction of TSG-6 by IFNγ and TNFα

**Fig. 4:**
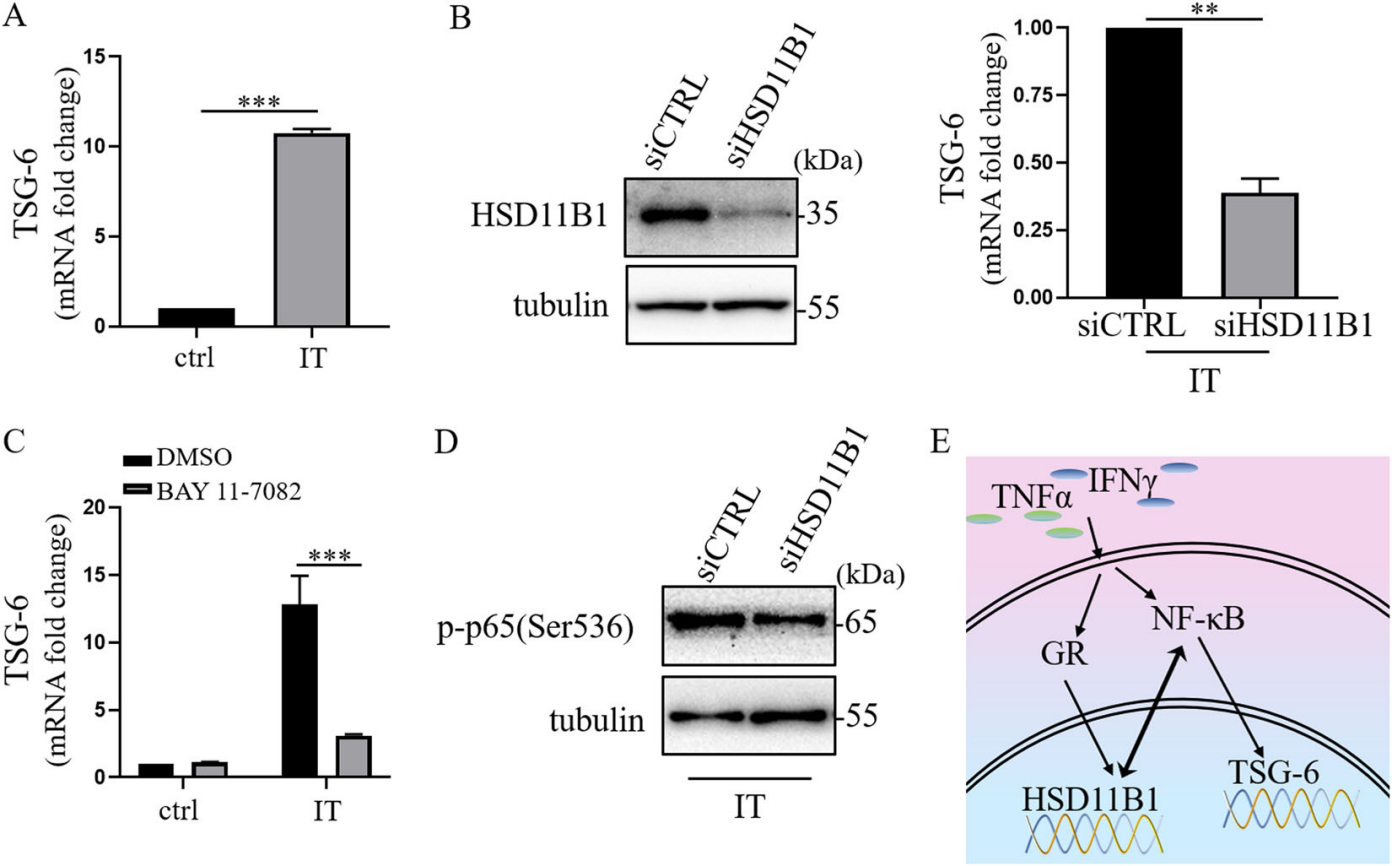
HSD11B1 mediated the expression of TSG-6 induced by IFNγ and TNFα via NF-κB. **a** TSG-6 in human UC-MSCs under IT (TNFα combined with IFNγ) stimulation was detected by real-time PCR. The histograms represent the mean values ± SEM from three independent experiments (*n* = 9). Asterisks indicate highly significant (***p < 0.001) differences (Student’ *t* test). Data are presented as fold change relative to ctrl human UC-MSCs. **b** Knockdown efficiency of siHSD11B1 was examined by Western blotting. TSG-6 in HSD11B1-depleted human UC-MSCs after IT stimulation was measured by real-time PCR. The histograms represent the mean values ± SEM from three independent experiments (*n* = 9). Asterisks indicate highly significant (**p < 0.01) differences (Student’ *t* test). Data are presented as fold change relative to siCTRL human UC-MSCs. **c** TSG-6 in human UC-MSCs under BAY 11-7082 and IT stimulation was analyzed by real-time PCR. The histograms represent the mean values ± SEM from three independent experiments (*n* = 9). Asterisks indicate highly significant (***p < 0.001) differences (ANOVA). Data are presented as fold change relative to DMSO-ctrl human UC-MSCs. **d** p-p65(Ser536) in HSD11B1-depleted human UC-MSCs under IT stimulation was detected by Western blotting. **e** The schematic overview of IFNγ and TNFα regulated HSD11B1 production and HSD11B1 regulated TSG-6 production in human UC-MSCs.

Like that of HSD11B1, the expression of TSG-6, an important immunoregulatory molecule, was significantly increased when stimulated by IFNγ and TNFα (Fig. 4a). We next tested whether there is link between HSD11B1 and TSG-6 by depleting HSD11B1 in human UC-MSCs. Interestingly, the expression of TSG-6 was greatly reduced after HSD11B1 depletion in IFNγ and TNFα-licensed human UC-MSCs (Fig. 4b), which strongly suggested that HSD11B1 was involved in the upregulation of TSG-6 by IFNγ and TNFα.

TSG-6 was identified as a transcriptional target of NF-κB^27^. As expected, the expression of TSG-6 in human UC-MSCs was downregulated when treated with NF-κB inhibitor, BAY 11-7082 (Fig. 4c). Furthermore, the phosphorylation of p65 in HSD11B1-depleted human UC-MSCs was decreased (Fig. 4d), which indicates that HSD11B1 may contribute to the maintenance of p65 signal pathway. Taken together, the data suggested that HSD11B1 might mediate the expression of TSG-6 through NF-κB.

## Discussion

The MSCs-based treatment of inflammatory diseases is being widely tested in preclinical and clinical studies. Because of their powerful anti-inflammatory property, it is safe to predict that MSCs therapy will be a potential treatment for diseases such as SARS and the current COVID-19, or novel coronavirus pneumonia, in which the damage caused by cytokine storms is hard to control. Previous studies had demonstrated that the immunosuppressive ability of MSCs is not innate, but, is induced by the proinflammatory cytokines, like IFNγ in combination with TNFα^12^. Thus, ironically, proinflammatory cytokines can lead to immunosuppression under certain circumstances^12^. In human UC-MSCs, these cytokines induce a dramatic upregulation in HSD11B1, an important enzyme that metabolizes and activates GC, which may enhance local anti-inflammatory effects of GC.

Stimulation of HSD11B1 activity following TNFα exposure has been described in early progenitor cells of the mesenchymal lineage and differentiated cells such as osteoblasts and adipocytes, particularly in cells of human origin^26,28,29^. These studies concluded that HSD11B1 induction by TNFα may enhance local GC generation and function. The regulation of HSD11B1 expression by IFNγ is less understood, although. IFNγ was reported to inhibit HSD11B1 expression in monocytes^30^. To our surprise, we observed that while IFNγ alone was unable to induce HSD11B1, it synergistically increased the expression of HSD11B1 when combined with TNFα. Our results thus revealed that the upregulation of HSD11B1 is exerted through the concerted action of TNFα and IFNγ in human UC-MSCs.

NF-κB was reported to positively regulate HSD11B1 expression in human synovial fibroblasts and human dermal fibroblasts^31^ and in adipocytes of diet-induced obese mice^32^. However, it was also shown to inhibit the expression of HSD11B1 in mouse skeletal muscle cells^33^. Our results with human UC-MSCs are consistent with those obtained with fibroblasts and adipocytes, which are presumably derived from MSCs. It should be noted that C/EBPα and C/EBPβ also serve as positive regulators of HSD11B1 P1 promoter regulator^34^. It is therefore reasonable to speculate that C/EBP may be involved in HSD11B1 regulation by IFNγ and TNFα. Further studies may reveal more species-, tissue- and context-specific factors in the regulation of HSD11B1.

GC is well documented to stimulate HSD11B1 expression and activity in most cell types. Consistently, HSD11B1 was found to be greatly upregulated by dexamethasone in human UC-MSCs, and this effect was abolished when RU486, a GC competitive GR inhibitor, was applied. Surprisingly, the HSD11B1 upregulation by IFNγ and TNFα was even further increased by RU486. In contrast, GR depletion led to a suppression of HSD11B1 in the same context. These seemingly contradictory results could be due to differential consequences of RU486 treatment and GR depletion. RU486 binding to GR may induce some conformational change of GR and further activate it. Indeed, the phosphorylation of GR at the Ser211, a marker of GR activation, was enhanced by RU486, which might lead to augmented HSD11B1 expression. These data collectively suggest that GR may play a structural role in the upregulation of HSD11B1 by IFNγ and TNFα.

We found that the upregulation of HSD11B1 by the inflammatory factors in human UC-MSCs contributes to their anti-inflammatory function. Depletion of HSD11B1 dramatically reduced the expression of TSG-6, suggesting that HSD11B1 is required for MSCs to acquire some of their anti-inflammatory properties.

Previous studies suggested that increasing HSD11B1 expression is a mechanism to initiate the process of inflammatory resolution through increased local GC generation in IL1β and lipopolysaccharide stimulation^35^. However, blocking GC binding to GR did not decrease the expression of TSG-6 induced by TNFα and IFNγ stimulation (data not show), which suggested that HSD11B1 could upregulated TSG-6 independent of GC. We confirmed TSG-6 as a target gene of NF-κB in human UC-MSCs. Furthermore, we noticed that the activity of NF-κB was repressed in HSD11B1-depleted MSCs. These results suggested that HSD11B1 mediated the induction of TSG-6 through NF-κB (Fig. 4e). The feedforward loop between NF-κB and HSD11B1 may augment the immunosuppressive function of MSCs under inflammatory conditions.

In conclusion, we demonstrated a critical role for GC metabolism in the acquisition of the immunosuppressive capability by human UC-MSCs exposed to proinflammatory cytokines. These findings may allow the better clinical utilization of MSCs.

## Materials and methods

### Isolation, expansion, and cultivation of human UC-MSCs

The human umbilical cord was obtained from the First Affiliated Hospital of Soochow University with the informed consent of the donor and approved for use by the ethical standards of the Ethics Committee of both Soochow University and the First Affiliated Hospital of Soochow University.

Human UC-MSCs were obtained from human umbilical cord using a previously described protocol^15^. Briefly, the umbilical cord was stored in sterilized glass bottles containing phosphate-buffered saline (PBS) for processing within 4 h. Then the umbilical cord was washed with PBS to remove blood, and then minced and seeded into cell culture dishes with low-glucose DMEM (HyClone, Neb, USA)-containing 10% fetal bovine serum (FBS) (Gibco, MA, USA), penicillin/streptomycin (10 units) (Thermo Fisher Scientific, MA, USA), and were incubated at 37 °C in 5% CO_2_ atmosphere. The medium was replaced for every 48 h with fresh medium. Upon reaching 80–90% confluency, cells were harvested using trypsin-EDTA and then used for experiments immediately or frozen and stored in liquid nitrogen. Human UC-MSCs were identified by the specific cell surface markers, including HLA-DR (−), CD45 (−), CD31 (−), CD34 (−), CD105 (+), CD29 (+), CD90 (+), CD73 (+), and CD44 (+). These MSCs were capable of differentiating into osteocytes and adipocytes under the respective differentiation conditions.

Human UC-MSCs were maintained in low-glucose DMEM (HyClone, USA), supplemented with FBS (10%) (Gibco, USA), penicillin/streptomycin (10 units) (Thermo Fisher, USA), 10 ng/ml human-bFGF (R&D, USA) and incubated at 37 °C in the presence of 5% CO_2_. The media were replaced for every 72 h and the cells were split twice a week. All cells were regularly tested to ensure they were mycoplasma-free. Cells were used before the 12th passage.

### Treatments of human UC-MSCs

Human UC-MSCs were cultured in 12-well plates, upon reaching 80–90% confluency, cells were washed with PBS and then treated with indicated stimulations. The stimulations were TNFα (eBioscience, MA, USA) or IFNγ (eBioscience, USA) alone or a combination of these two cytokines (10 ng/ml each) for 24 h; TNFα (10 ng/ml) and IFNγ (10 ng/ml) combined with BAY 11-7082 (5 μM) (Selleck, WA, USA) or DMSO (Sigma, MA, USA) for 24 h; TNFα (10 ng/ml) and IFNγ (10 ng/ml) combined with RU486 (3 μM) (Selleck, USA) or DMSO for 24 h; Dexamethasone (10 ng/ml) (Sigma, USA) combined with RU486 (3 μM) or DMSO for 24 h.

### Real-time PCR

Total RNA was extracted from cell lysates according to the manufacturer’s instructions using Trizol reagent (Thermo Fisher Scientific, USA). First-strand cDNA synthesis was performed using PrimeScript^™^ RT Master Mix (TaKaRa Biotech, Dalian, China) according to the manufacturer’s instructions. The total reaction volume of 10 μL was comprised of 1 ng cDNA, 3 μL DNAase/RNAse-free water (TaKaRa Biotech, China), 1 μL primers (GENEWIZ, Suzhou, China), 5 μL SYBR qPCR SuperMix plus (with ROX) (Novoprotein, Shanghai, China). After pipetting the reaction mixture into a 384-well plate, real-time PCR was running by QuantStudio 6 Flex (Applied Biosystems, MA, USA). mRNA levels were calculated referring to β-actin as a housekeeping gene. To compare the expression of each gene in the different treatment conditions, the fold change of expression was calculated using the equation 2^−ΔΔCt^ where ΔΔCt = ΔCt (treated) − ΔCt (control), ΔCt (treated) = [Ct (target gene) − Ct (β-actin)], ΔCt (control) = [Ct (target gene) − Ct (β-actin)]. Real-time PCR was performed using the following primers β-actin-F 5′-TTGCCGACAGGATGCAGAAGGA-3′, β-actin-R 5′-AGGTGGACAGCGAGGCCAGGAT-3′, HSD11B1-F 5′-AGCAGGAAAGCTCATGGGAG-3′, HSD11B1-R 5′-CCACGTAACTGAGGAAGTTGAC-3′, TSG-6-F 5′-TTTCTCTTGCTATGGGAAGACAC-3′, TSG-6-R 5′-GAGCTTGTATTTGCCAGACCG-3′. The total amount of mRNA was normalized to endogenous β-actin mRNA.

### Western blotting

Total cellular proteins were extracted by RIPA buffer (Beyotime, Shanghai, China) containing PMSF (Beyotime, China) and phosphatase inhibitors (Roche, NJ, USA). Protein samples were separated on a 10% sodium dodecyl sulfate-polyacrylamide gel, and separated proteins were electroblotted onto polyvinylidene difluoride membranes. The membranes were blocked for 2 h with tris-buffered saline containing Tween 20 (TBST) with 5% albumin bovine serum (Amresco, OH, USA) at room temperature with gentle shaking. Then incubated the membranes with primary antibodies HSD11B1 (ab39364, abcam, MA, USA), tubulin (2128S, Cell Signaling Technology, MA, USA), p-p65 (Ser536) (3033S, Cell Signaling Technology, USA), p65 (8242S, Cell Signaling Technology, USA), p-GR (Ser211) (4161S, Cell Signaling Technology, USA), or GR (12041S, Cell Signaling Technology, USA) for 10 h at 4 °C with gently shaking. After washing 3 times with TBST for 5 min each, the membranes were incubated with horse radish peroxidase-conjugated rabbit secondary antibodies (7074S, Cell Signaling Technology, USA) for 1 h at room temperature with gentle shaking. After incubation, membranes were harvested with NcmECL Ultra Kit (NCM biotech, Suzhou, China) according to the manufacturer’s instructions and the signal was detected by Ultra-sensitive automatic imaging analysis system (ProteinSimple, CA, USA). Tubulin was used as an internal control.

### Transfections with siRNA

Human UC-MSCs were plated on 12-well plate at the density of 50% per well and were treated with 1 µl INTERFERin® reagent (PolyPlus-transfection, Illkirch, France) along with 1 μl GR, p65 or HSD11B1 siRNA (GenePharma, Shanghai, China). The same amount of control siRNA (GenePharma, China) was also transfected into MSCs as control according to the manufacturer’s protocols. After 48 h, cells were washed twice with PBS, and then cells were treated with indicated stimulations. The efficiency of transection was monitored by Western blotting. p65 siRNA sequences were 5′-CCCUAUCCCUUUACGUCAUTT-3′ and 5′-AUGACGUAAAGGGAUAGGGTT-3′. GR siRNA sequences were 5′-GAUGUAAGCUCUCCUCCAUTT-3′ and 5′-AUGGAGGAGAGCUUACAUCTT-3′. HSD11B1 siRNA sequences were 5′-GCAGGAAGAUCCUGGAAUUTT-3′ and 5′-AAUUCCAGGAUCUUCCUGCTT-3′. Control siRNA sequences were 5′-UUCUCCGAACGUGUCACGUTT-3′ and 5′-ACGUGACACGUUCGGAGAATT-3′.

### Statistical analysis

Each experiment was performed in biological triplicate each time and repeated independently at least three times. The data are presented as mean ± standard error of the mean. Statistical significance was analyzed using Student’s *t* test, analysis of variance test calculation using GraphPad Prism 8 (GraphPad Software Inc., CA, USA), *p* values < 0.05 were considered statistically significant. *p* < 0.05 is denoted as *, *p* < 0.01 as **, *p* < 0.001 as ***, *p* < 0.0001 as ****.
